# Impact of hemolysis on multi-OMIC pancreatic biomarker discovery to derisk biomarker development in precision medicine studies

**DOI:** 10.1038/s41598-022-05152-8

**Published:** 2022-01-24

**Authors:** Richard Searfoss, Punit Shah, Kennedy Ofori-Mensa, Valerie Bussberg, Vladimir Tolstikov, Bennett Greenwood, Hongyan Li, Kris Richardson, Gregory M. Miller, Corinne DeCicco, Elder Granger, Leonardo O. Rodrigues, Eric M. Grund, A. James Moser, Rangaprasad Sarangarajan, Niven R. Narain, Michael A. Kiebish

**Affiliations:** 1 BERG, Framingham, MA 01701 USA; 2 Beth Israel Deaconess, Harvard Medical School, Boston, MA 02215 USA

**Keywords:** Bioinformatics, Proteomic analysis

## Abstract

Cancer biomarker discovery is critically dependent on the integrity of biofluid and tissue samples acquired from study participants. Multi-omic profiling of candidate protein, lipid, and metabolite biomarkers is confounded by timing and fasting status of sample collection, participant demographics and treatment exposures of the study population. Contamination by hemoglobin, whether caused by hemolysis during sample preparation or underlying red cell fragility, contributes 0–10 g/L of extraneous protein to plasma, serum, and Buffy coat samples and may interfere with biomarker detection and validation. We analyzed 617 plasma, 701 serum, and 657 buffy coat samples from a 7-year longitudinal multi-omic biomarker discovery program evaluating 400+ participants with or at risk for pancreatic cancer, known as Project Survival. Hemolysis was undetectable in 93.1% of plasma and 95.0% of serum samples, whereas only 37.1% of buffy coat samples were free of contamination by hemoglobin. Regression analysis of multi-omic data demonstrated a statistically significant correlation between hemoglobin concentration and the resulting pattern of analyte detection and concentration. Although hemolysis had the greatest impact on identification and quantitation of the proteome, distinct differentials in metabolomics and lipidomics were also observed and correlated with severity. We conclude that quality control is vital to accurate detection of informative molecular differentials using OMIC technologies and that caution must be exercised to minimize the impact of hemolysis as a factor driving false discovery in large cancer biomarker studies.

## Introduction

The current healthcare ecosystem is rapidly evolving toward deploying precision medicine strategies for increasing optimal stratification of patients to improve clinical outcomes. These actions will predominantly focus on the use of molecular, digital, and clinical biomarkers that will characterize patients on multiple dimensions of phenotypic presentation. Standardization of quality parameters governing sample collection are important to ensure accuracy and reproducibility of potential discoveries ultimately easing translation back into the clinic. Molecular markers, whether genetic, proteomic, lipidomic or metabolomic, hold tremendous promise to deconvolute the biological presentation of patients. The composition of adaptive biological molecules (proteins, lipids, and metabolites) can be significantly influenced by patient demographics, pharmacological agents, and sample handling processes which can hinder potential biomarker discovery and development.

Hemolysis represents a common sample processing outcome and can be due to handling, but also disease etiology rendering red blood cells (RBC) more labile for lysis. Hemolysis can occur for a variety of reasons and leads to the release of free hemoglobin into blood collection samples^1^. Due to some medical conditions, or as the result of taking certain medications, this breakdown of RBC’s can be increased. Hemolysis has the potential to drastically alter the observed proteome of buffy coat samples due to contamination of hemoglobin and other high-abundance proteins seen in RBC. RBC are mainly comprised of hemoglobin and carbonic anhydrase-1, contributing 97% and 1% of the entire RBC proteome, respectively^2^. The buffy coat fraction of whole blood has been observed to be < 1% of the blood by volume^3^. As a result, even minor contamination of RBC into the other fractions, or increased hemolysis due to medical reasons, can increase the concentration of hemoglobin and carbonic anhydrase-1 and potentially impact the observed proteome.

Guidelines governing omics analysis of clinical samples have been developed over the past decade as the use of such platforms has been broadly adopted in R&D and clinical trial assessments^4,5^. This includes standardized sample preparation approaches and techniques, quality controls, and the recommended size of cohorts required to ensure statistical significance of potential findings. However, protocols for quality controls regarding sample collection are deficient. Several key challenges have already been demonstrated in using biofluids for biomarker discovery, such as chemical modifications of proteins or sample degradation during storage. Further, utilizing plasma and serum, which is often employed for convenience of collection, exhibits a wide dynamic range of protein concentrations, making the identification of low abundance potential biomarkers all the more challenging. One potentially impactful occurrence that should be included is the effect of hemolysis, which can directly contribute to both aforementioned challenges.

Herein, we performed mass spectrometry based lipidomics, metabolomics and proteomics analysis of plasma and serum from over 420 individuals in pancreatic biomarker clinical trial. Buffy coat samples were only subjected to proteomics analysis based on the sample amount obtained. A subset of samples obtained were impacted by hemolysis resulting in contamination of the matrix of interest. A comprehensive assessment of expressional patterns of proteins, lipids and metabolites was performed to identify hemolytic contamination in these samples. The proteome of buffy coat was most impacted, resulting in expressional changes of proteins originating from red blood cells. The use of markers impacted by hemolysis should considered with caution for exploration as biomarkers.

## Method

### Study design

There were 420 patients enrolled in this study: 224 males and 196 females. These fell into one of the five categories as follows; healthy volunteers: 33, patients with pancreatitis: 113, early pancreatic cancer: 67, local pancreatic cancer: 115 and metastatic pancreatic cancer: 92. Informed consent was obtained from all the participants in the study NCT02781012.

All experimental protocols were approved by WCG institutional review board. Research use of the samples was conducted in accordance with the terms outlined within the informed consent form and the terms set forth therein and with the tenets of the Declaration of Helsinki and its later amendments or comparable ethical standards.

### Sample collection

Whole blood samples were collected via venipuncture into EDTA tubes. All samples were processed and frozen at − 80 °C within 3 h of the blood draw. The plasma fraction was separated using centrifugation at 1200×g for 10 min at room temperature and was aliquoted into separate tubes and frozen. During centrifugation, the buffy coat layer also separated from the red blood cells. The buffy coat layer was collected and diluted with 8 mL RPMI buffer, transferred into a 50 mL Leucosep tube, and centrifuged at 1200×g for 10 min at room temperature to separate the buffy coat layer further from the red blood cells. Buffy coat was washed three times with PBS and pelleted to remove solution. Finally, the buffy coat was resuspended in 200 µL of PBS and split between two tubes before being frozen at − 80 °C. A separate vial of blood was collected for serum sample collection in serum separator tubes and was left at room temperature for 30–45 min to allow for the clot to form. Serum separator tubes were then centrifuged at 1200×g for 10 min at room temperature. Separated serum was aliquoted and frozen at − 80 °C.

### Detection of hemolysis

**Figure 1:**
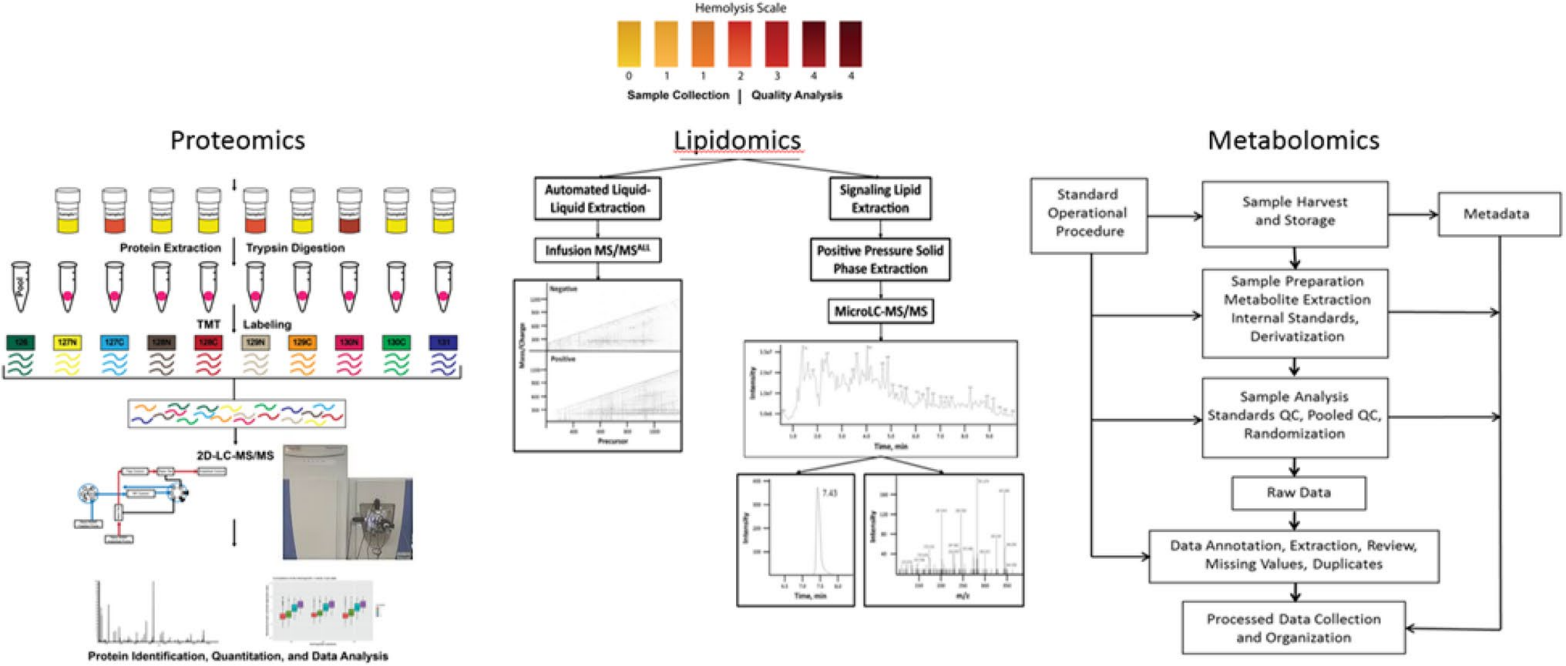
Workflow of the methods used to study the impact of Hemolysis. Initially, clinical samples were assigned a hemolysis score of 0–4 following the hemolysis scale color legend. In proteomics, plasma and serum were filtered and depleted of the top 14 most abundant proteins, and buffy coat cells were lysed. Proteins were extracted and digested with trypsin before being labeled with TMT 10-Plex. TMT-labeled peptides were analyzed using 2D LC–MS/MS platform and quantified using Proteome Discoverer v1.4. In lipidomics, structural lipids were extracted via liquid/liquid extraction method on an automated Hamilton Robotics STARlet system. Extracted lipids were analyzed via direct injection electrospray ionization TOF–MS. Further, mediator lipids were acidified and extracted using SPE. Eluted lipids were dried and resuspended for LC–MS analysis. In metabolomics, metabolites were extracted in organic conditions and analyzed using gas chromatography–mass spectrometry (GC/MS), reversed-phase liquid chromatography–mass spectrometry (RP-LC/MS), and hydrophilic interaction chromatography–liquid chromatography–tandem mass spectrometry (HILIC-LC/MS/MS). Post-processing of data included inspection and merging.

Upon receipt, all samples were accessioned and qualitatively assigned a colorimetric hemolysis score of 1–3 for plasma and serum and 0–4 for buffy coat following the color scale in Fig. 1^7^. A score of zero was reserved for buffy coat samples appearing clear to opaque white when buffy coat cells were most pure. Given the natural yellowish appearance of plasma and serum, a score of zero was never given, and a score of 1 was considered most pure.

### Ethics approval and consent to participate

This study was IRB approved and all patients consented to participate.

### Consent for publication

All authors consent to publication.

## Proteomics

### Protein extraction

65 μL of raw plasma/serum was filtered through a pre-wet 0.22 μm cellulose acetate spin filter. 40 μL of the filtered plasma/serum was pipetted onto another pre-wet 0.22 μm cellulose acetate spin filter and combined with 20 μL of 80 mg/mL lipid removal agent (LRA). The mixture was placed on a shaker for 30 min and then centrifuged. The resulting filtrate was roughly 40 μL in volume and was combined with 120 μL of Agilent Buffer A. The sample was then loaded into vials and placed on the Agilent 1260 series HPLC, and the top 14 abundant proteins were depleted using the Multi-Affinity Removal Column 14 from Agilent. The depleted samples were collected into vials and protein concentration was determined using the Bradford Assay.

Buffy coat samples were lysed with a lysis buffer containing 5 M Urea, 50 mM Tris–HCl pH 8.3, 0.1% SDS, 1% Protease and Phosphatase Inhibitor Cocktail, and Optima LC/MS Water. 100 μL of lysis buffer was added to each sample and mixed by pipetting up and down, and then the whole sample was immediately transferred out of the sample vial and into a 1.5 mL Eppendorf tube. Each sample was sonicated with four 3-s pulses at 20% amplification to fully lyse the cells. Sonicated samples were centrifuged at 17,000×g for 10 min, and the supernatant was then used in the Bradford Assay to determine the protein concentration.

### Trypsin digestion

Extracted proteins were trypsin digested as previously described^7^. In brief, proteins were reduced with 10 mM Tris(2-carboxyethyl) Phosphine (TCEP) and alkylated with 18.75 mM iodoacetamide before being precipitated in acetone overnight and digested with trypsin the next day.

### TMT labeling of peptides

10-Plex TMT (Tandem Mass Tag) reagents were used to label peptides from all samples, allowing relative quantitation (Thermo Fisher Scientific). For buffy coat samples, a reference sample was created by pooling an aliquot of peptides from each individual sample. For plasma and serum, a reference sample was purchased from BioIVT and prepped alongside clinical samples (Westbury, CT, USA). Peptides (20 µg) from each of the samples were dissolved in 20 µL of 200 mM triethylammonium bicarbonate (TEAB), pH 8.5 solution, and mixed with 20 µL of TMT reagent that was freshly dissolved in 256 µL of anhydrous acetonitrile, LCMS grade. Channel 126 was used for labeling the pooled reference sample in all matrices analyzed in this study. After 1 h incubation at RT, the reaction was quenched by adding 8 µL 5% hydroxylamine. Peptides labeled by different TMT reagents were then mixed, dried and desalted on C18 Spin columns. Desalted peptides were dried in a vacuum centrifuge and stored at − 20 °C until LC–MS/MS analysis.

### Mass spectrometry

LC–MS/MS analysis was performed using a Waters nanoAcquity 2D LC system coupled to a Thermo Q Exactive Plus MS. TMT-labeled MPs were resolved over 12 fractions, 90-min gradient per fraction, and fractionated using two-dimensional reversed-phase chromatography prior to MS analysis. In the first dimension, Buffer A is 20 mM Ammonium formate pH 9.5 and Buffer B is 100% Acetonitrile. Peptides are separated on a basic reverse phase compatible column BEH C18 trap column, 300 μM × 50 mm, particle size 5 μM (waters) into 12 fractions with %B 7.4, 10.8, 12.6, 14, 15.3, 16.7, 18.3, 20.4, 23.5, 50, 65 and wash. In the second dimension, peptides were separated in a 90 min gradient from 5%B to 85%B.

The eluting peptides were sprayed into the mass spectrometer using electrospray ionization and a data dependent Top 15 acquisition method was used to fragment candidate ions. Full MS survey scans were collected at a resolution of 35,000, scan range of 400–1800 Thompsons (Th; Th = Da/z), followed by MS/MS scans at a resolution of 35,000 with a 1.2 Th isolation window. Only ions with a + 2 to + 4 charge were considered for isolation and fragmentation. Data was searched using Proteome Discoverer 1.4 using SEQUEST and Mascot algorithms and uniprot database.

## Metabolomics

### Metabolomics analysis

Plasma and serum samples for metabolomics analysis were prepared as previously described^8–12^. Metabolite extraction was achieved using a mixture of isopropanol, acetonitrile, and water at a ratio of 3:3:2 v/v. Extracts were divided in to three parts: 75 µL for gas chromatography combined with time-of-flight high-resolution mass spectrometry, 150 µL for reversed-phase liquid chromatography coupled with high-resolution mass spectrometry, and 150 µL for hydrophilic interaction chromatography with liquid chromatography and tandem mass-spectrometry, and analyzed as previously described^8–12^. We used the NEXERA XR UPLC system (Shimadzu, Columbia, MD, USA), coupled with the Triple Quad 5500 System (AB Sciex, Framingham, MA, USA) to perform hydrophilic interaction liquid chromatography analysis, NEXERA XR UPLC system (Shimadzu, Columbia, MD, USA), coupled with the Triple TOF 6500 System (AB Sciex, Framingham, MA, USA) to perform reversed-phase liquid chromatography analysis, and Agilent 7890B gas chromatograph (Agilent, Palo Alto, CA, USA) interfaced to a Time-of-Flight Pegasus HT Mass Spectrometer (Leco, St. Joseph, MI, USA). The GC system was fitted with a Gerstel temperature-programmed injector, cooled injection system (model CIS 4). An automated liner exchange (ALEX) (Gerstel, Muhlheim an der Ruhr, Germany) was used to eliminate cross-contamination from the sample matrix that was occurring between sample runs. Quality control was performed using metabolite standards mixture and pooled samples, applying the methodology previously described^13–16^. A quality control sample containing a standard mixture of amino and organic acids purchased from Sigma-Aldrich as certified reference material, was injected daily to perform an analytical system suitability test and to monitor recorded signals day to day reproducibility as previously described^8–12^. A pooled quality control sample was obtained by taking an aliquot of the same volume of all samples from the study and injected daily with a batch of analyzed samples to determine the optimal dilution of the batch samples and validate metabolite identification and peak integration. Collected raw data was manually inspected, merged and normalized by the sample median. Metabolite identification was performed using in house authentic standards analysis. Metabolite annotation was used utilizing recorded retention time and retention indexes, recorded MS^n^ and HRAMS^n^ data matching with METLIN, NIST MS, Wiley Registry of Mass Spectral Data, HMDB, MassBank of North America, MassBank Europe, Golm Metabolome Database, SCIEX Accurate Mass Metabolite Spectral Library, MzCloud, and IDEOM databases.

## Lipidomics

### Structural lipidomic analysis

A cocktail of deuterium-labeled and odd chain phospholipid standards from diverse lipid classes was added to 25 µL of thawed serum or plasma. Standards were chosen to represent each lipid class and were prepared at concentrations proportional to the endogenous amount per sample matrix to provide the most accurate quantitation and dynamic range for each lipid species. 4 mL chloroform:methanol (1:1, v/v) was added to each sample and the lipid extraction was performed as described. Lipid extraction was automated using a customized sequence on a Hamilton Robotics STARlet system (Hamilton, Reno, NV, USA) to meet the high-throughput requirements. Lipid extracts were dried under nitrogen and reconstituted in 68 µL chloroform:methanol (1:1, v/v). Samples were flushed with nitrogen and stored at − 20 °C. Samples were diluted 50-fold in isopropanol:methanol:acetonitrile:water (3:3:3:1, by volume) with 2 mM ammonium acetate in order to optimize ionization efficiency in positive and negative modes. Electrospray ionization-MS was performed on a TripleTOF^®^ 5600^+^ (SCIEX, Framingham, MA, USA), coupled to a customized direct injection loop on an Eksigent microLC200 system (SCIEX) as described^17,18^.

### Mediator lipidomic analysis

A mixture of deuterium-labeled internal standards was added to aliquots of 100 µL serum or plasma, followed by 3× volume of sample of cold methanol (MeOH). Samples were vortexed for 5 min and stored at − 20 °C overnight. Cold samples were centrifuged at 14,000×*g* at 4 °C for 10 min, and the supernatant was then transferred to a new tube and 3 mL of acidified H_2_O (pH 3.5) was added to each sample prior to C18 SPE columns (Thermo Pierce) and performed as described^19^. The methyl formate fractions were collected, dried under nitrogen, and reconstituted in 50 µL MeOH:H_2_O (1:1, v/v). Samples were transferred to 0.5 mL tubes and centrifuged at 20,000×*g* at 4 °C for 10 min. Thirty-five µL of supernatant were transferred to LC–MS vials for analysis using the BERG LC–MS/MS mediator lipidomics platform as described.

## Data analysis

### Missing data and omics normalization

Omics data with missing values in more than 85% of samples were considered below detection limit, and therefore removed from further analysis. For proteomics data, processing began by merging data collected across multiple batches (a.k.a. “MPs”) to create a single data frame containing all features measured in any of the collected samples. Omics datasets were normalized according to a median centering and variance scaling approach applied across samples using custom R scripts^20,21^. Proteomics data were corrected for multiple MP batches using an empirical Bayesian framework, ComBat^22,23^. Briefly, this method performed location and scale adjustments based on estimated batch effect parameters per protein and returned a corrected dataset for further analysis. Missing proportions of proteins were determined by comparing total number of proteins identified across all samples of each type, and number of proteins not identified in each sample relative to total identified proteins 3647, groups by hemolysis score of 0, 1, 2 and 3.

### Differential expression

The normalized data from above were used to perform differential expression in each Omic type between hemolysis groups (e.g.; Hemolysis group 3+ vs. 0 within Proteomics). Differentially expressed Omics features were identified by using Limma version 3.42.2 in an R36 environment. The control Hemolysis Group for Proteomics was 0 and 1 for Lipidomics and Metabolomics. The limma::lmFit() function was used to produce a fitted model containing coefficients and residuals for each omics comparison. Subsequently, a moderated t-statistic of differential expression was computed for each comparison for each protein using the limma::eBayes() function. A Benjamini and Hochberg multiple correction was applied to results from each differential analysis which can be observed in the adj.p.val column for each differential expression summary table (Supplementary Tables S1–S7).

### Covariate analysis

To determine if the features of age, sex or disease status could be confounding the comparisons made in the differential expression analyses a Pearson’s Chi-Square test was performed between hemolysis score and sex or disease status. For age, data was binned into 8 groups, from 20 to 90 in increments of 10 and a Pearson’s Chi-Square test performed with hemolysis score. In each case no *p* value was lower than 0.10 of the compared features. As a result, the differential expression models were not adjusted for age, sex, or disease status.

## Results

### Workflow, design and summary

**Figure 2:**
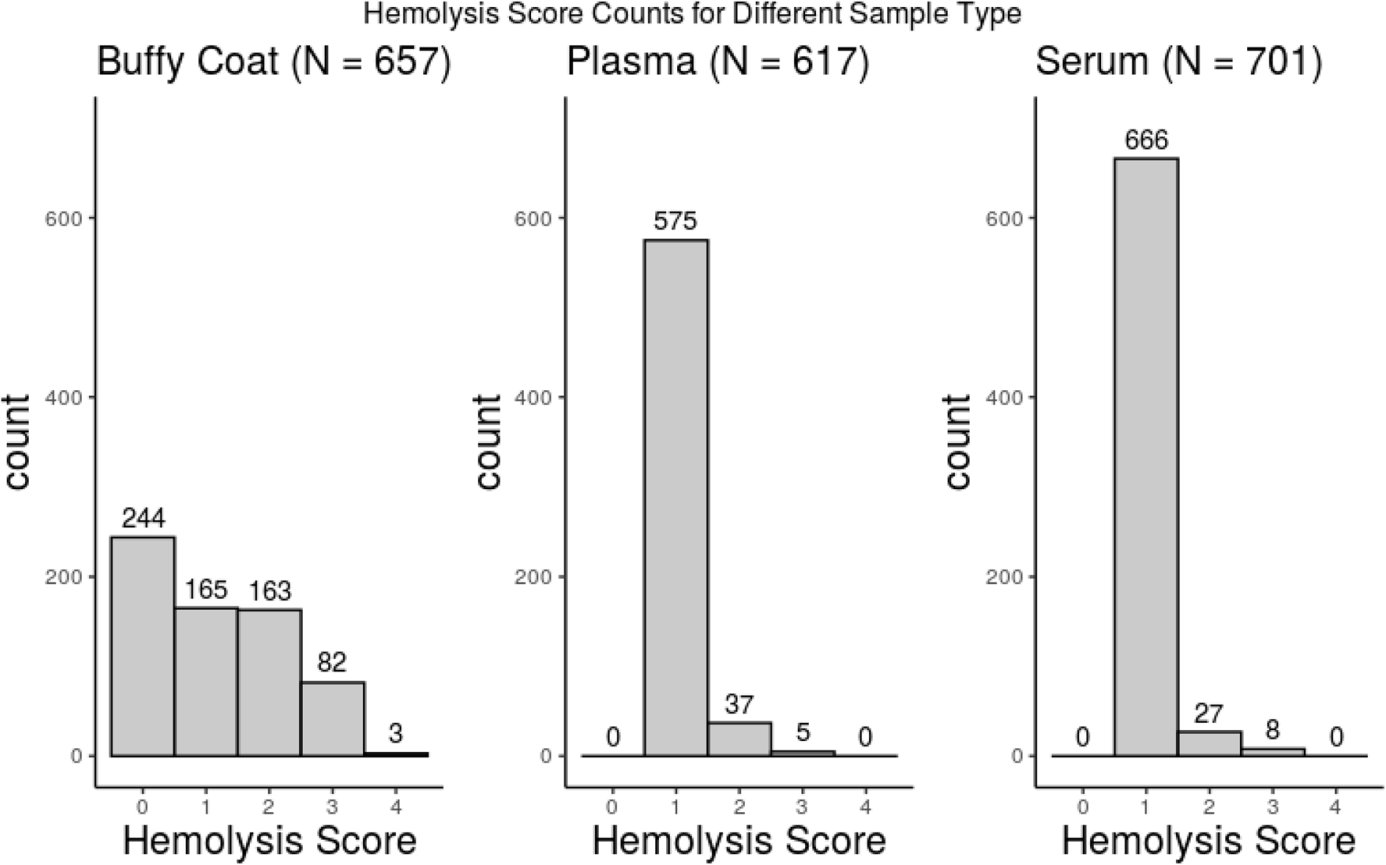
Hemolysis score distribution of sample count buffy coat (N = 657), Plasma (N = 617), and Serum (N = 701) samples included in this study.

To evaluate the impact of hemolysis on biomarker discovery utilizing a multi-omics platform, we compared proteins, lipids, and metabolites identified across plasma, serum, and buffy coat samples (proteomics only) acquired from 420 non-diseased and pancreatic cancer patients. Workflow of Proteomics, Lipidomic and Metabolomic analysis is shown in Fig. 1. A hemolysis score was recorded for each sample, ranging from 0 to 4 for buffy coat and 1–3 for plasma and serum. A summary of the distribution of hemolysis scores within each sample type can be found in Fig. 2. Buffy coat yielded the largest hemolyzed samples 37.1% #0, 25.1% #1, 24.8% #2, 12.4% #3, 0.4% #4 hemolysis. Protocol of isolation of buffy coat from blood may be one of the major reasons for the large number of contaminated buffy coat samples.

In proteomics, 7302, 1971, 2146 proteins were identified and quantified in buffy coat, serum and plasma, respectively, using TMT labeling and 2D online LC–MS/MS. After filtering the data for proteins that have less than 85% missing values, a total of 3647, 453, and 492 proteins in buffy coat, serum and plasma, respectively, were obtained and used for further analysis. In lipidomics, 1318 structural lipids and 106 mediator lipids were identified and quantified after data filtration for analysis in plasma and serum samples. In metabolomics, a total of 514 and 508 metabolites were identified and quantified in plasma and serum samples, respectively, after data filtering and kept for further analysis.

### Differentially expressed metabolites and lipids

Lipidomics analysis revealed no significant changes in lipid expression for mediator lipidomics data when comparing samples with hemolysis scores of 2+ to 1 in both plasma and serum. However, for structural lipidomics analysis, 5 lipids were found to be downregulated, and 2 lipids upregulated in plasma (Supplemental Table S4), and 14 lipids were downregulated, and 11 lipids upregulated in serum (Supplemental Table S5; Table 1). More profound effects were seen in the metabolomics data. When comparing samples with hemolysis scores of 2+ to 1, a total of 51 metabolites were found to be downregulated and a total of 25 upregulated due to hemolysis in plasma (Supplemental Table S6; Table 1). For the same comparison in serum, 93 metabolites were downregulated and 21 were upregulated due to hemolysis (Supplemental Table S7; Table 1). A summary of these results can be found in Supplemental Table 1.

**Table 1:** Differentially expressed species due to hemolysis

Matrix	Proteomics			Signaling lipidomics		Structural lipidomics		Metabolomics	
	Buffy coat	Plasma	Serum	Plasma	Serum	Plasma	Serum	Plasma	Serum
Downregulated species	148	0	1	0	0	5	14	29	48
Upregulated species	240	19	10	0	1	2	11	16	20

### Missingness

**Figure 3:**
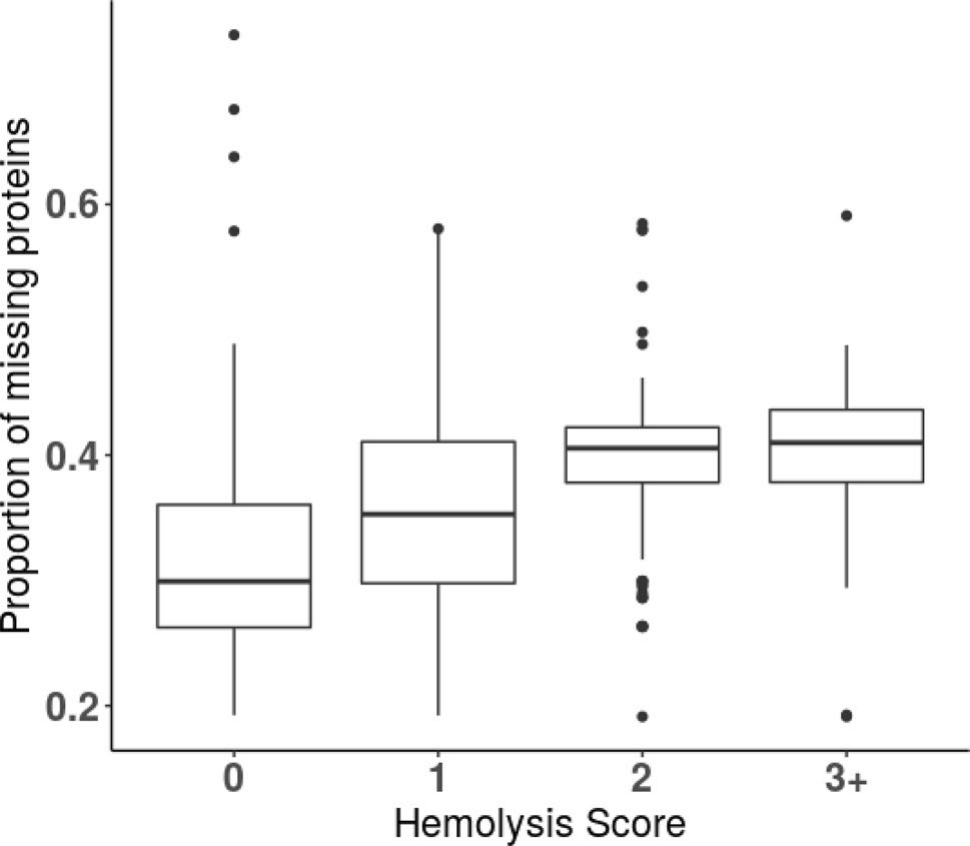
Boxplots visualizing relative proportion of missing proteins. Missing proteins were determined by comparing total number of proteins identified across all samples of each type, and number of proteins not identified in each sample relative to total identified proteins 3647, groups by hemolysis score of 0, 1, 2 and 3.

A subset of samples with the lowest hemolysis score was created, in this case, a score of 0 for buffy coat samples and a score of 1 for plasma and serum samples. This subset was used to filter the proteins, and only the proteins that have < 85% missing values were kept in the full proteomics data. The missing proportions of proteins for each sample were computed, and samples were then grouped by hemolysis score of 0:244 samples, score 1:165 samples, score 2:163 samples, score 3+: 85 samples in buffy coat (Fig. 2). The boxplots clearly indicate that as the hemolysis score of a sample increases, the number of proteins that are identified across the set within the sample decreases, and the medians of proportions of missing proteins are 0.299, 0.353, 0.406, 0.410 for the groups with hemolysis score 0, 1, 2, 3+, respectively (Fig. 3). This can be explained by an increase in the signal derived from the more abundant hemoglobin proteins contributed from the lysed red blood cells, suppressing the signal of the less abundant proteins and changing the dynamic range of the protein content that would ideally be identified from samples with little to no hemolytic contamination.

### Differential expressed proteins

**Figure 4:**
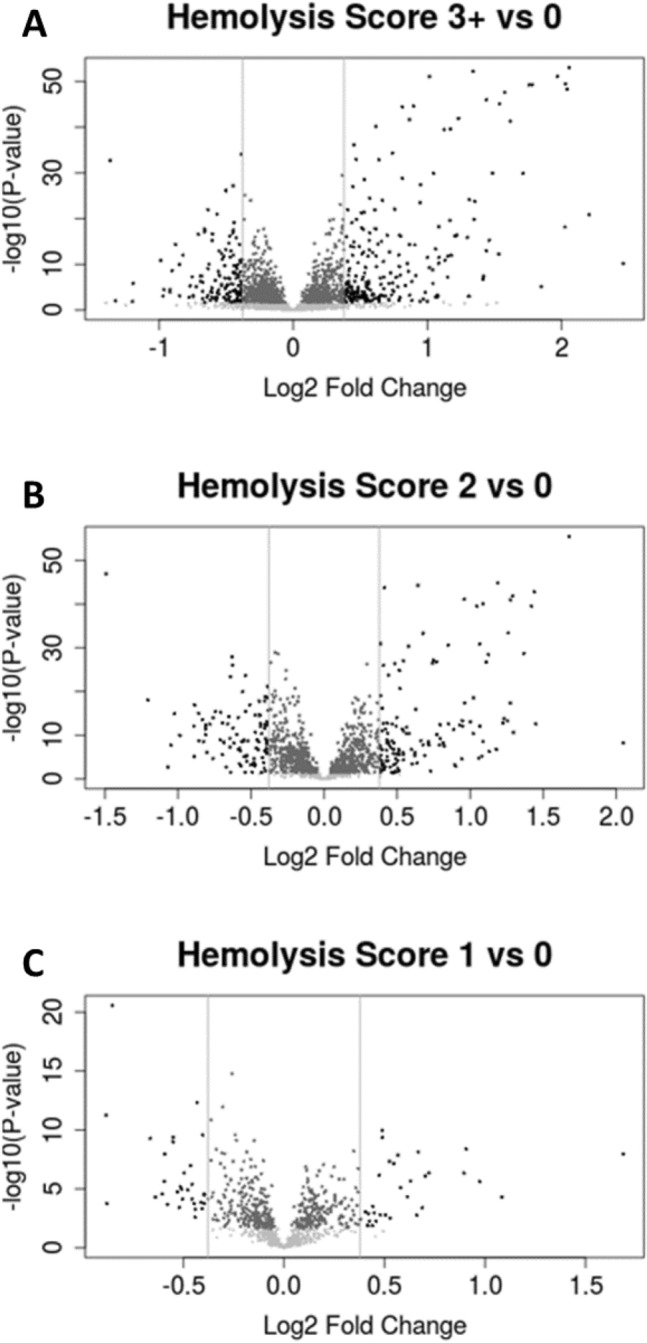
Volcano plots showing a comparison of protein expression between 3 versus 0, 2 versus 0 and 1 versus 0. The expression of protein ratio to the QCP was exhibited as Log2 fold and compared to − Log10 of *p* value. Significant proteins required minimum 1.3 fold-change difference and maximum *p* value of 0.05.

To assess the effect of hemolysis on relative protein expression in buffy coat, comparisons between hemolysis groups were performed as shown by volcano plots (Fig. 4A). Overall, 657 samples were included in this analysis. A total of 3647 proteins were identified when assessing the differentially expressed proteins between samples with a score of 0 versus 1 (Fig. 4A), with 76 differently expressed proteins downregulated and 48 proteins upregulated at a 1.3 fold change threshold and an adjusted *p* value of 0.05. Comparing samples with a score of 0 versus 2 (Fig. 4B), a total of 701 proteins were consistently identified across all samples, with 173 proteins differently expressed proteins downregulated and 196 proteins upregulated at a 1.3 fold change threshold and an adjusted *p* value of 0.05. Lastly, we compared samples with a score of 0 versus 3+ Fig. 4C, and a total of 592 proteins were consistently identified across all samples, with 148 proteins differently expressed proteins downregulated and 240 proteins upregulated at a 1.3 fold-change threshold and an adjusted *p* value of < 0.05 (Supplemental Table S1). Hemolysis not only impacted the proteins identified but also impacted the quantitation of the differentially expressed proteins.

Further, comparisons between samples with no visual hemolysis (scores of 0 for buffy coat, scores of 1 for plasma and serum) were made to samples with visual hemolysis (scores of 1–3+ for buffy coat, scores of 2+ for plasma and serum). Differential expression of proteins was observed using volcano plots shown in Fig. 4, using a threshold of 1.3 fold change with a corresponding adjusted *p* value of < 0.05 to be considered differentially expressed. Overall, 148 proteins were found to be downregulated and 240 upregulated in buffy coat (Supplemental Table S1). A total of 19 proteins in plasma and 11 proteins in serum were found to be upregulated in the same comparison (Supplemental Tables S2 and S3).

### Impact of hemolysis on hemoglobin

**Figure 5:**
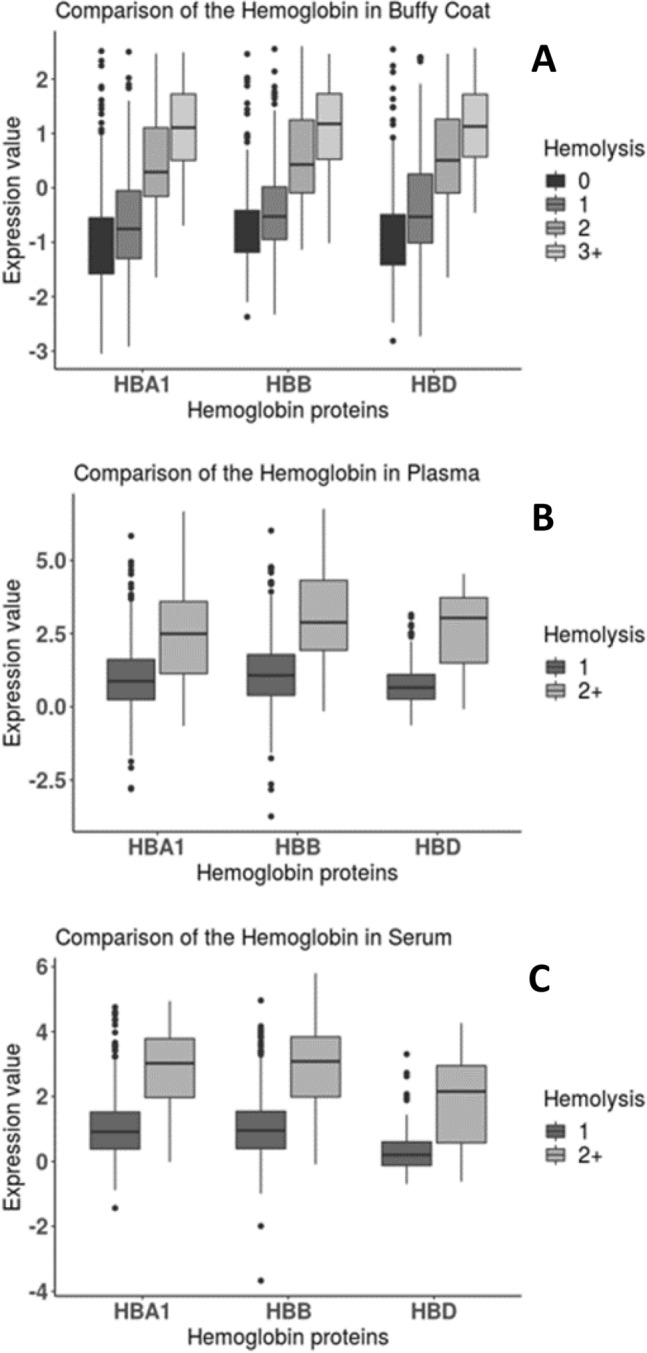
Expression of hemoglobin proteins in (**A**) buffy coat, (**B**) plasma, and (**C**) serum (HBA1 = Hemoglobin Subunit Alpha, HBB = Hemoglobin Subunit Beta, HBD = Hemoglobin Subunit Delta) as a relative measure of hemolysis. (**A**) HBA1 protein expression comparison between hemolysis score of 0 versus 1 in buffy coat with adjusted *p* value of 0.0014, comparison between 0 versus 2 with adjusted *p* value of 1.29e^−38^ and comparison between 0 versus 3 with adjusted *p* value of 2.29e^−47^. HBD protein comparison between 0 versus 1 with adjusted *p* value of 1.40E^−05^, comparison of 0 versus 2 with adjusted *p* value of 6.23e^−44^ and comparison of 0 versus 3 with adjusted *p* value of 7.28E^−49^. HBB protein comparison between 0 versus 1 with adjusted *p* value of 0.0005, comparison of 0 versus 2 with adjusted *p* value of 1.13e^−40^ and comparison of 0 versus 3 with an adjusted *p* value of 2.62e^−47^. Samples were grouped into hemolysis score 1 and 2+ for plasma and serum due to low number of score 3 and 4 samples in these matrices. (**B**) For plasma shows HBA1 protein comparison of hemolysis score 1 versus 2 with adjusted *p* value of 6.65E^−09^, HBB protein comparison 1 versus 2 with adjusted *p* value of 4.24e^−10^, and HBD protein comparison of 1 versus 2 with adjusted *p* value of 0.00027. **C** For serum shows HBA1 protein comparison 1 versus 2 with adjusted *p* value of 4.16e^−22^, HBB protein comparison 1 versus 2 with adjusted *p* value of 1.15e^−23^, and HBD protein comparison of 1 versus 2 with adjusted *p* value of 3.56e^−09^.

To study hemolysis via protein identification and relative quantitation, we assessed the expression of hemoglobin subunit alpha (HBA1), hemoglobin subunit beta (HBB), and hemoglobin subunit delta (HBD) across all sample types and grouped by hemolysis score within each sample type. Hemolysis is generally classified as the lysis of RBC in circulation or during sample preparation, and as hemoglobin is one of the most abundant proteins in red blood cells, the hemoglobin expression increases due to hemolysis (Fig. 5A) and increased stepwise with increasing hemolysis score. A similar pattern was seen in both plasma and serum, with lower levels observed in samples with a hemolysis score of 1, and significantly higher levels observed in samples scored 2+ (Fig. 5B, C).

**Figure 6:**
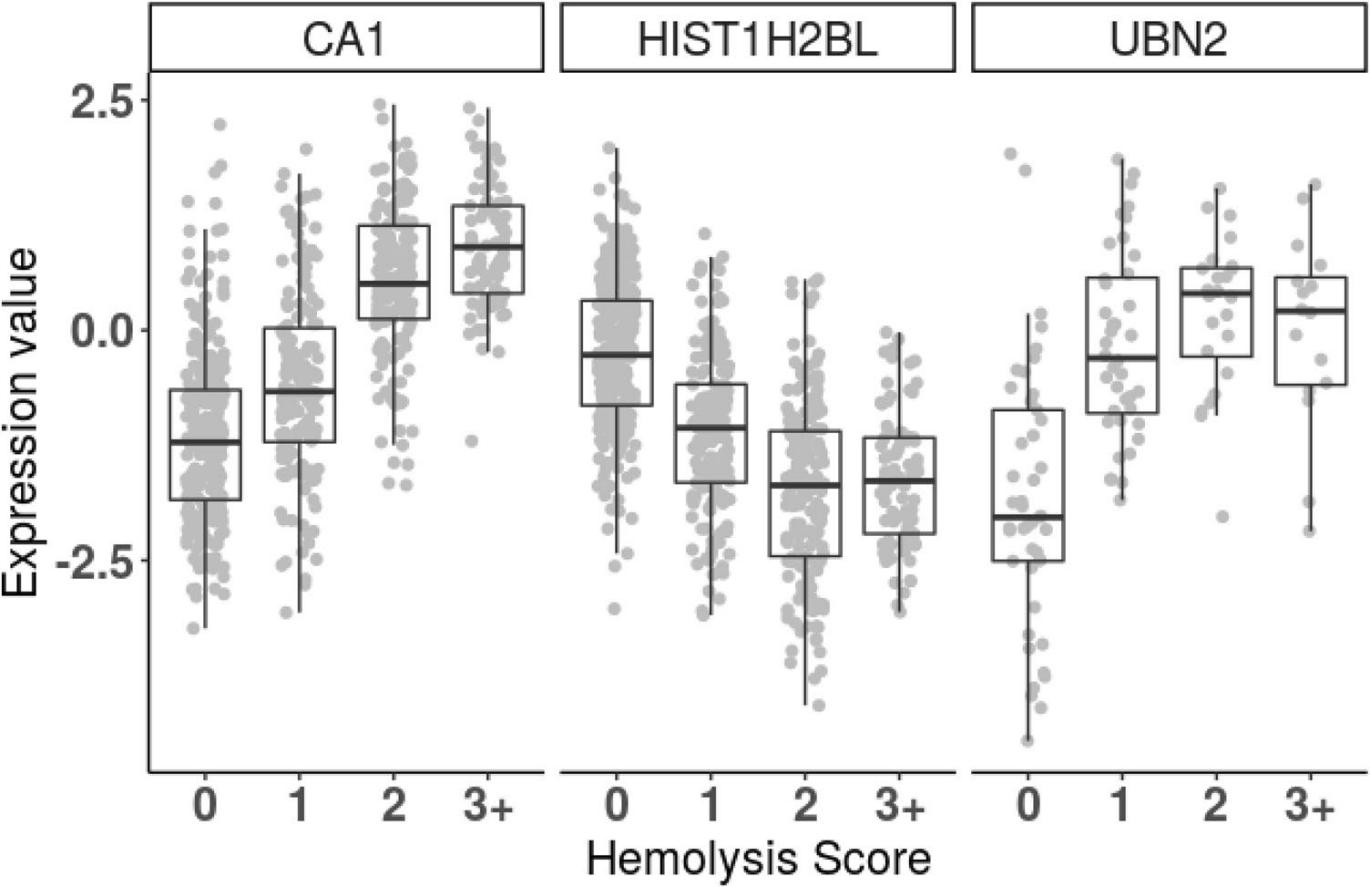
is a boxplot of buffy coat proteins expressions of (CA1 = Carbonic anhydrase, HIST1H2BL = Histone H2B type 1-L, UBN2 = Ubinuclein-2) that were identified as significantly differentially expressed proteins in comparison to their Hemolysis score of 0, 1, 2 and 3+. Expression values are log2 ratio to the reference sample. CA1 protein comparison between 0 versus 1 with an adjusted *p* value of 5.54E^−07^, comparison of 0 versus 2 with adjusted *p* value of 1.56e^−53^ and comparison of 0 versus 3 with adjusted *p* value of 3.27e^−50^. UBN2 protein comparison between 0 versus 1 with adjusted *p* value of 4.57e^−07^, comparison of 0 versus 2 with adjusted *p* value of 2.88e^−08^ and comparison of 0 versus 3 with adjusted *p* value of 5.42e^−05^. H2BC13 protein comparison between 0 versus 1 with adjusted *p* value of 2.72e^−18^, whereas comparison of 0 versus 2 with adjusted *p* value of 2.60e^−45^ and comparison of 0 versus 3 with adjusted *p* value of 3.12e^−31^.

We also assessed the expression of carbonic anhydrase (CA1), histone H2B type 1-L (HIST1H2BL), and ubinuclein-2 (UBN2) (Fig. 6). CA1 is another major protein found in RBC's and is responsible for processing carbon dioxide in the body. The expression of CA1 is low in samples classified with a hemolysis score of 0, and increases similar to the hemoglobin protein expression with increasing hemolysis score (Fig. 6). HIST1H2BL and UB2 are both nuclear proteins whose identification is expected in buffy coat samples and not from red blood cells. HIST1H2BL and UBN2 expression follow the expected result, with higher expression in samples with hemolysis score of 0 and lower expression with increasing hemolysis score (Fig. 6), indicating signal suppression of these proteins as a result of hemolysis.

## Discussion

Translation of biomarkers into clinical practice requires the comprehensive understanding of the impact of sample handling to avoid false discovery of processing markers rather than disease associated biomarkers. Adaptive omic technologies such as proteomics, lipidomics, and metabolomics demonstrate tremendous promise associating the patient phenotypic with causal biology but are also significantly impacted by red blood cell contamination in plasma, serum, or buffy coat. In the current study, we uncovered that buffy coat was the most significantly affected by hemolysis in a prospective biomarker study investigating pancreatic cancer and at-risk populations. A Pearson’s Chi-Square test of independence was performed between hemolysis score and disease status, age, or gender, in a univariate manner (Supplemental Table S8 to S12). None of these potential confounders demonstrated a significant impact on hemolysis score and the incidence of hemolysis was independent of disease conditions but did influence detection and quantification of analytes.

Contamination by proteins found in RBC from hemolysis has also been demonstrated in red blood cell storage in an occurrence known as storage lesions. Storage lesions are progressive changes in the morphology, biochemistry, and function of RBC during storage that result in changes in the viability of the RBC and accumulation of contaminating proteins and cells. These changes in RBC ultimately lead to hemolysis, and consequently, a release of the cytosolic contents into solution^1,24^. A study observing changes in the protein distribution of RBC supernatant over a storage period identified appreciable increases in proteins, including carbonic anhydrase 1 and 2 (CA1 and CA2), peroxiredoxin-1 and -2 (PRDX1 and PRDX2), and catalase, as well as others, due to hemolysis of RBC over time in these storage lesions^25^. Similarly, our findings also conclude these proteins to be contaminants in plasma, serum, and buffy coat due to hemolysis that may occur in vivo or during sample processing.

The identification of proteins in a sample depends on the dynamic range of the proteins. Identifying less abundant proteins in a sample via LC–MS/MS analysis is challenging at low concentrations as current mass spectrometry capabilities allow for identification over a range of 3–4 orders of magnitude^26^. Hemolysis increases the hemoglobin content in the sample of interest. Given that hemoglobin accounts for 97% of the composition of RBC’s, with carbonic anhydrase accounting for another 1%, this can create significant suppression of signal of low abundant proteins in the biofluid of choice for a proteomic study^27^. In proteomics, sample quantitation is performed using equal volume of fluid or equal concentration of protein content. In this study the equal concentration of proteins was used for semi-quantitation, supplemented by Tandem Mass Tags for protein quantitation. The general hypothesis is that the samples are identical with minor changes. Quantitation of proteins is impacted due to hemolysis which leads to an increase in concentration of red blood cell proteins. As contamination increases, the proportion of proteins of interest in the sample decreases and can lead to inaccurate quantitation and false discovery of the biomarkers. Hemolyzed samples should be avoided in omics studies to minimize data analysis variability and data interpretation errors. The use of differentially expressed species (Supplemental Table S1) as biomarkers of disease in any study should be viewed with caution due to hemolysis. For instance, carbonic anhydrase-1 has been demonstrated as a biomarker in serum for prostate cancer^28^. Further, peroxiredoxin-2 was identified as a biomarker in a panel of proteins from plasma for Anderson–Fabry disease^29^. While this may in fact be the case, careful consideration should be taken into sample quality while testing to avoid false positives, and analysis should be performed to conclude these proteins had little to no contribution to their signal from sample handling issues or hemolysis.

In clinical settings, omics analysis on serum, plasma or buffy coat samples requires caution while handling samples to avoid hemolysis. Following a set protocol is required when collecting and handling samples and any deviation in sample handling needs to be recorded. In some cases, even after all sample handling precautions have been taken, hemolysis may still occur due to underlying biological factors. In these scenarios, various methods can be used for data analysis to minimize the impact of contamination of proteins like hemoglobin. One approach is to ignore any contributing red blood cell proteins as a biomarker, if considered as contamination. A second approach is to use proteins such as hemoglobin or carbonic anhydrase to normalize the data and specifically normalize only non-red blood cell containing proteins. This can minimize the impact due to hemolysis in quantitation. However, any attempt that might minimize this effect may not completely negate the impact due to hemolysis. A third approach is to move towards equal volume quantitation compared to equal concentration quantitation, however, this might require technical advancements in instrumentation and technology. Identification of contaminating proteins cannot be avoided, and expression of those protein rise with the increase in the hemolysis score. Sophisticated LC–MS/MS technology, biochemical procedures for sample preparation and advance bioinformatics tools need to be used for omics analysis in precision medicine. Using stringent purification procedures are of key importance in using blood samples for identification and application of biomarkers. A limitation of this study is accounting for the degree to which potential confounding factors influence the outcome. Furthermore, replication of these findings will be essential for determining their utility.

This study comprehensively assessed omics variables significantly impacted by the increase in hemolysis score in buffy coat and plasma/serum. Differences were identified that were associated with increasing hemolysis score, including missingness of proteins identified. Integration of lipidomics, metabolomics and proteomics data provided an expanded, comprehensive insight of the impact of hemolysis. Overall, our results will serve as a comprehensive resource to the biomarker community in the field of blood analysis. Diagnostic applications will be able to leverage these proteins, lipids and metabolites identified as hemolytic contamination for future biomarker studies.

## Supplementary Information

Supplementary Information.

## Data Availability

The datasets used and/or analyzed during the current study are available from the corresponding author on reasonable request.
